# Repeated transcranial photobiomodulation improves working memory of healthy older adults: behavioral outcomes of poststimulation including a three-week follow-up

**DOI:** 10.1117/1.NPh.9.3.035005

**Published:** 2022-09-27

**Authors:** Xiujuan Qu, Lexuan Li, Xiaohan Zhou, Qi Dong, Hanli Liu, Hesheng Liu, Qin Yang, Ying Han, Haijing Niu

**Affiliations:** a Beijing Normal University, IDG/McGovern Institute for Brain Research, State Key Laboratory of Cognitive Neuroscience and Learning, Beijing, China; b University of Texas at Arlington, Department of Bioengineering, Arlington, Texas, United States; c Medical University of South Carolina, Department of Neuroscience, Charleston, South Carolina, United States; d Xuanwu Hospital of Capital Medical University, Department of Neurology, Beijing, China; e Hainan University, School of Biomedical Engineering, Haikou, China; f Beijing Institute for Brain Disorders, Center of Alzheimer’s Disease, Beijing, China; g National Clinical Research Center for Geriatric Diseases, Beijing, China

**Keywords:** neuromodulation, transcranial photobiomodulation, older adults, working memory, repeated stimulation

## Abstract

**Significance:**

Decline in cognitive ability is a significant issue associated with healthy aging. Transcranial photobiomodulation (tPBM) is an emerging non-invasive neuromodulation technique and has shown promise to overcome this challenge.

**Aim:**

This study aimed to investigate the effects of seven-day repeated tPBM, compared to those of single tPBM and baseline, on improving N-back working memory in healthy older adults and to evaluate the persistent efficacy of repeated tPBM.

**Approach:**

In a sham-controlled and within-subject design, 61 healthy older adults were recruited to participate in a longitudinal study involving an experimental baseline, seven days of tPBM treatment (12 min daily, 1064-nm laser, $250\;\mathrm{mW}/{\mathrm{cm}}^{2}$) in the left dorsolateral prefrontal cortex and three weeks of follow-ups. Behavioral performance in the N-back (N=1,2,3) was recorded poststimulation during the baseline, the first and seventh days of the tPBM session, and the three weekly follow-ups. A control group with 25 participants was included in this study to rule out the practice and placebo effects. The accuracy rate and response time were used in the statistical analysis.

**Results:**

Repeated and single tPBM significantly improved accuracy rate in 1- and 3-back tasks and decreased response time in 3-back compared to the baseline. Moreover, the repeated tPBM resulted in a significantly higher improvement in accuracy rate than the single tPBM. These improvements in accuracy rate and response time lasted at least three weeks following repeated tPBM. In contrast, the control group showed no significant improvement in behavioral performance.

**Conclusions:**

This study demonstrated that seven-day repeated tPBM improved the working memory of healthy older adults more efficiently, with the beneficial effect lasting at least three weeks. These findings provide fundamental evidence that repeated tPBM may be a potential intervention for older individuals with memory decline.

## 1 Introduction

Over the last few decades, a large number of older adults have suffered from cognitive decline,^1^^–^^3^ and it is predicted that the number will still increase in the coming years. The decline in cognitive ability, particularly in memory, is a significant problem of healthy aging and is associated with neurobiological changes that preferentially affect the frontal lobe of the cerebral cortex.^2^^–^^4^ Practical strategies for preventing or delaying cognitive decline in older individuals have become a priority and mainly attempt to improve the functioning of the frontal lobe.

Transcranial photobiomodulation (tPBM), an emerging non-invasive neuromodulation technology, has shown promising potential for cognitive improvements. In general, the technique entails projecting low-power and high-fluence light from lasers or light-emitting diodes in the red to near-infrared range (620 to 1100 nm) onto the cerebral cortex. The light that penetrates cerebral tissue is absorbed by cytochrome c oxidase (CCO) in the mitochondrial electron transport chain, which leads to enhancement of mitochondrial redox metabolism^1^^,^^2^^,^^5^ and increased production of adenosine triphosphate and ultimately benefits brain functions.^6^^–^^8^

Recent studies have shown that a few minutes of tPBM over the frontal cortex could significantly improve cognitive outcomes, such as memory^7^ and executive function^9^ in young adults. Meanwhile, other studies have also documented the beneficial effects of repeated tPBM treatment on improving the cognitive capacity of patients with mental or neurological disorders.^10^^–^^12^ For example, Disner et al.^10^ revealed that repeated tPBM treatment (8 min per session, two sessions 48 h apart) could augment the benefits of attention bias modification in adult participants with depression. Chao et al.^11^ also discovered that the application of repeated tPBM treatment (three times per week and lasting 12 weeks) could significantly improve the cognitive performance of older patients with mild-to-moderate dementia. However, since the findings from young adults and patients cannot be easily extrapolated to older populations, little is known about tPBM-induced cognition enhancement in healthy older adults.

Recently, two studies have provided preliminary evidence indicating that both single and repeated tPBM treatment could benefit cognition performance in healthy older adults. For instance, Chan et al.^4^ found that given older adults with a single tPBM treatment (7.5 min) exhibited immediate improvements in inhibitory control and mental flexibility. Using repeated tPBM treatment (8 min per session, one session per week for five weeks), Vargas et al.^13^ found that both the reaction time of the vigilance task and the accuracy of the delayed match-to-sample (DMS) memory task were significantly enhanced. Moreover, a recent model-based dosimetry study demonstrated that repeated tPBM stimulations might lead to a cumulative light exposure sufficient to neuromodulate a larger proportion of the target tissues than what was estimated from a single stimulation.^12^ This speculation is particularly instructive for the tPBM treatment of older adults since an overall decrease of energy deposition caused by tPBM was found with the increasing thickness of extracerebral tissues and increasing age;^14^ therefore, repeated sessions, rather than a single one may be associated with more promising clinical effectiveness. As such, whether repeated tPBM treatment leads to a greater improvement in cognition (e.g., working memory) for healthy older adults than single tPBM treatment deserves further exploration. Moreover, it is also unknown whether and how long the efficacy of tPBM treatment lasts.

In the present study, we examined the effects of repeated tPBM treatment applied to the left dorsolateral prefrontal cortex (DLPFC), compared to single and baseline, on N-back working memory performance in healthy older adults. The persistent effects of repeated tPBM treatment on working memory were also examined via a follow-up design. We hypothesized that repeated tPBM treatment would enhance the working memory of healthy older adults more effectively than single tPBM treatment and that the effects of repeated tPBM treatment would be sustained within a period after stimulation.

## 2 Material and methods

### 2.1 Participants

A total of 319 healthy older adults responded to Xuanwu Hospital’s recruitment advertisement at Capital Medical University. After screening and clinical evaluation, which included a medical history interview, a neurological examination, a battery of neuropsychological tests, and routine laboratory tests,^15^ 86 participants (age: 50 to 77 years, 64.76±5.81  years, 69 females) were eligible to take part in the experiment. Written informed consent was obtained from all participants prior to the study. The study’s inclusion criteria were as follows: (1) subjects had to be 50 to 79 years old, right-handed, and speak Mandarin; (2) subjects needed to be free of cognitive impairments; and (3) subjects had to be able to complete the study. The exclusion criteria were as follows: (1) subjects that had current primary psychiatric diagnoses such as severe depression or anxiety; (2) subjects that had other neurological conditions (e.g., cerebrovascular disease, brain tumors, Parkinson’s disease, encephalitis, or epilepsy); and (3) subjects that had other diseases that could cause cognitive decline [e.g., thyroid dysfunction, severe anemia, syphilis, or human immunodeficiency virus (HIV)]. The institutional review board at Xuanwu Hospital in Capital Medical University approved this study.

### 2.2 Procedures

**Fig. 1:**
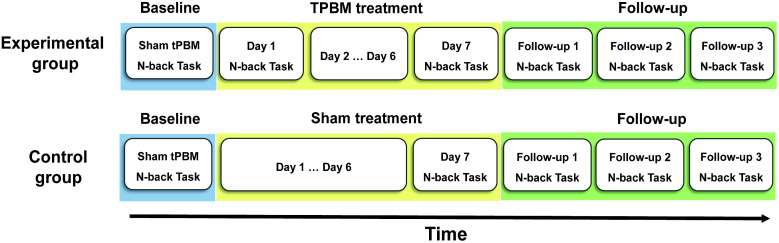
Illustration of the experimental design. This design included an experimental group and a control group, and each of them involved three sessions: baseline session, treatment session (one-week daily tPBM treatment for the experimental group and sham treatment for the control group), and follow-up session. N-back working memory tasks were recorded during the baseline, treatment, and follow-up sessions, respectively.

This was a sham-controlled, single-blinded, randomized within-subject study. The experimental group included 61 healthy older adults, who underwent a baseline session, one week of tPBM treatment session, and a follow-up session accordingly (Fig. 1). Specifically, during the baseline period, all participants first received a 12-min sham treatment to the left DLPFC, followed by an N-back (N=1,2,3) working memory task. During the tPBM sessions, the participants first received a 12-min tPBM treatment once a day for seven successive days, and then the N-back working memory task was administered after the end of the tPBM treatment session on the first (day 1, i.e., single tPBM treatment) and seventh (day 7, i.e., repeated tPBM treatment) days. Behavioral performance at the baseline, day 1, and day 7 were compared to demonstrate the effect of repeated tPBM treatment on working memory improvement. During the follow-up session, the N-back working memory task was administered once a week for three successive weeks (follow-ups 1, 2, and 3). Likewise, behavioral performance during each follow-up was evaluated to demonstrate the persistent effect of repeated tPBM treatment on working memory.

A control group (n=25) was also included in this current study to rule out both the practice and placebo effects. The participants underwent a similar procedure as the experimental group but received sham treatment during the one-week repeated tPBM treatment session (Fig. 1). Behavioral tests were conducted at baseline, day 7, and the weekly follow-up session for three weeks.

### 2.3 Transcranial Photobiomodulation

Transcranial near-infrared laser stimulation was applied with a continuous wavelength of 1064 nm (CNI laser-MIL-N-1064, China). The laser aperture diameter was 4.16 cm, and the output power was 3.4 W, resulting in irradiance of $0.25\;W/{\mathrm{cm}}^{2}$. These parameters are harmless to the human body and have been found to improve cognition and mental health in previous studies.^9^^,^^13^^,^^16^ The tPBM and sham treatment began after informed consent was obtained from each participant. During two treatments, both participants and the laser experimenters were required to wear protective eyewear, and participants were instructed to keep their eyes closed. For tPBM treatment, each participant received 12 min of active tPBM on the left DLPFC. For sham treatment, the operating procedures were the same as those of tPBM treatment, except that the laser was on only for the first 30 s and then turned off. None of the participants reported an uncomfortable feeling of heat on their skin with the current parameters.

### 2.4 Working-Memory Performance (N-Back Tasks)

We used a widely recognized N-back (N=1,2,3) task to assess the effect of working memory performance in healthy older adults after tPBM treatment. The digital N-back paradigm was adopted since it is much easier for older adults to view and respond to than letters. The task included three conditions of varying cognitive loads where participants were presented with one white number at a time centered on a black screen. In the “1-back” condition, participants indicated whether the number presented in the current trial was identical to the number presented in the immediately preceding trial by pressing a “4” button with their right hand. During the “2-back” condition, participants were instructed to respond when the number shown in the current trial was identical to the number presented two steps earlier. Similarly, during the “3-back” condition, participants were instructed to indicate whether the number shown in the current trial corresponded to the number displayed three steps earlier. Each cognitive load condition was presented as a block lasting 45 s. Each block consisted of a 5 s instruction screen followed by 20 trials (trial = stimulus displayed for 500 ms + 1500-ms blank screen, inter-stimulus interval = 2 s). Each cognitive load block was presented three times in pseudorandom order for a total of 9 blocks (three 1-back blocks, three 2-back blocks, and three 3-back blocks). The task began and ended with a 15-s crosshair for visual fixation, and a 10-s crosshair fixation separated each block, resulting in a total task run of 510 s (8 min 30 s). The participants underwent a practice version before tPBM treatment to ensure they understood it before completing the formal experiments.

The accuracy rate and response time were used to evaluate cognitive performance under different working memory loads (1-, 2-, and 3-back). The accuracy rate was calculated as the number of correct responses divided by the total number of responses, and the response time was estimated as the average response time before button press in trials with different working memory loads.

### 2.5 Data Overview

All participants tolerated tPBM treatment well, and no adverse effects were reported during or after tPBM treatment. Some participants dropped out of the study halfway for various personal reasons, which finally led to sample sizes of n=61 (baseline), n=57 (day 1), n=47 (day 7), n=41 (follow-up 1), n=39 (follow-up 2), n=36 (follow-up 3) for the experimental group, and n=25 (baseline), n=19 (day 7), n=15 (follow-up 1), n=15 (follow-up 2), n=12 (follow-up 3) for the control group.

### 2.6 Data Analysis

Participants with an accuracy rate or response time over three standard deviations away from the group mean were excluded as outliers in each session. Participants who completed both baseline and tPBM treatment sessions were included in the analysis on the effect of repeated tPBM treatment on working memory performance. Similarly, those who completed all baseline/day 7 and three follow-ups were included in the analysis of the persistent effects of repeated tPBM treatment. Demographic information, including age, sex, and years of education of the participants in both the experimental group and control group are included in Table 1.

**Table 1:** The demographic characteristics of the participants. Age and education were presented as the range of minimum-maximum (mean ± SD).

	Total	Experiment group	Control group
Sample (n)	81	61	25
Age (years)	65.52 ± 5.82	65.60±5.41	65.32 ± 6.80
Sex (F/M)	48/14	34/9	14/5
Education (years)	12.74 ± 2.86	12.81 ± 2.91	12.58 ± 2.81

### 2.7 Statistical Analysis

To investigate the effect of repeated tPBM treatment on working memory performance, we conducted a two-way repeated-measures analysis of variances (ANOVA) on accuracy rate and response time with stimulation type (baseline, day 1, and day 7) and cognitive load (1-back, 2-back, and 3-back) as factors. If the effect of stimulation type × cognitive load was significant, the main effect of stimulation type under each cognitive load was then investigated using a one-way repeated-measures ANOVA. If the main effect of stimulation type was significant, pairwise t-tests between any two of these stimulation types were subsequently carried out under each cognitive load to explore whether repeated tPBM-induced working memory performance was improved compared with that of baseline or single tPBM treatment.

To investigate whether the effect of repeated tPBM treatment persisted and how long it persisted, we conducted a one-way repeated-measures ANOVA with time (i.e., day 7/baseline and three follow-ups) as a repeated-measure factor for the cognitive loads with significant improvements in the preceding analysis. Day 7 and the three follow-ups were compared, and if the main effect of time was insignificant, it could be concluded that the effects of repeated tPBM treatment did not significantly decline during the follow-up session. In addition, an identical analysis was conducted on the data from the baseline and the three follow-ups. If the main effect of time was significant, pairwise t-tests between baseline and each follow-up were subsequently carried out, and if the behavioral performance was still significantly higher than baseline, it could be indicated that the improvement induced by repeated tPBM treatment can be persisted.

Mauchly’s test was conducted to evaluate the sphericity of the data before performing repeated-measures ANOVA. As the sphericity assumption was violated, Greenhouse–Geisser values were reported. A significance level of p<0.05 was used for all statistical comparisons. All analyses were carried out using SPSS version 26 (IBM SPSS Statistics, New York). Furthermore, for the control group, a permutation test was conducted to examine the difference of behavioral performance between follow-up and day 7 as well as follow-up and baseline (p<0.05, 1000 permutations).

## 3 Results

### 3.1 Effects of Repeated tPBM Treatment on Working Memory Performance

**Fig. 2:**
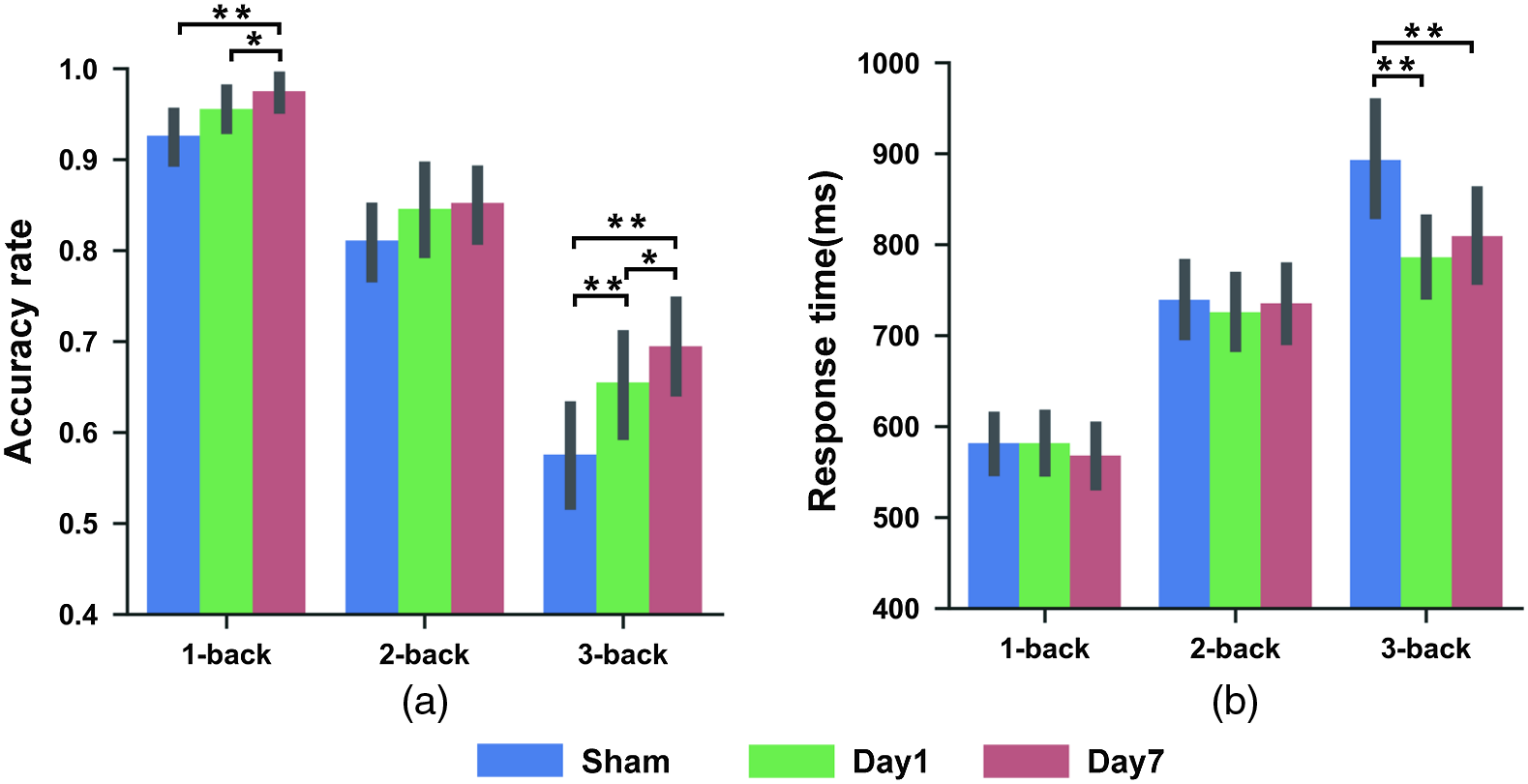
Working memory performance of the experimental group during baseline, single (i.e., day 1), and repeated (i.e., day 7) tPBM treatment. The (a) averaged accuracy rate and (b) response time are shown for varying levels of cognitive load. The error bars indicate the standard error of the mean. *p<0.05, **p<0.01.

The two-way ANOVA revealed significant interaction effects between stimulation type (i.e., baseline, day 1, and day 7) and cognitive loads (i.e., 1-, 2-, and 3-back) for both accuracy rate [F(3.06,125.29)=3.20, p=0.03] and response time [F(3.22,131.95)=8.23, p=0]. For accuracy rate [Fig. 2(a)], a significant main effect of stimulation type was observed in the 1- and 3-back conditions [one-way repeated-measures ANOVA; 1-back: F(1.50,61.42)=6.94, p=0.00; 3-back: F(2,82)=15, p=0]. Further statistical analysis of these two conditions revealed that both single [t(41)=−3.26, p=0] and repeated [t(41)=−5.18, p=0] tPBM treatment resulted in a significant improvement on the 3-back condition, but repeated tPBM treatment led to a greater improvement than single tPBM [t(41)=−2.13, p=0.04]. The improvement on the 1-back condition mirrored this pattern, except that the comparison between single and baseline only showed a marginally significant difference [baseline versus single: t(41)=−2.00, p=0.05; baseline versus repeated: t(41)=−3.18, p=0.00; single versus repeated: t(41)=−2.30, p=0.03].

For response time [Fig. 2(b)], there was a significant main effect of stimulation type only in the 3-back condition [F(2,82)=11.66, p=0]. Specifically, significantly decreased response time [Fig. 2(b)] was found after single [t(41)=4.61, p=0] and repeated tPBM treatment [t(41)=3.22, p=0] as compared to baseline, but no significant difference between single and repeated tPBM treatment [t(41)=−1.16, p=0.25].

### 3.2 Evaluation of the Persistent Effects of Repeated tPBM Treatment on Working Memory

Based on the above results (Fig. 2), we further investigated the persistent effects of repeated tPBM treatment on 1- and 3-back since the behavioral performance was significantly improved after stimulation on these two conditions.

**Fig. 3:**
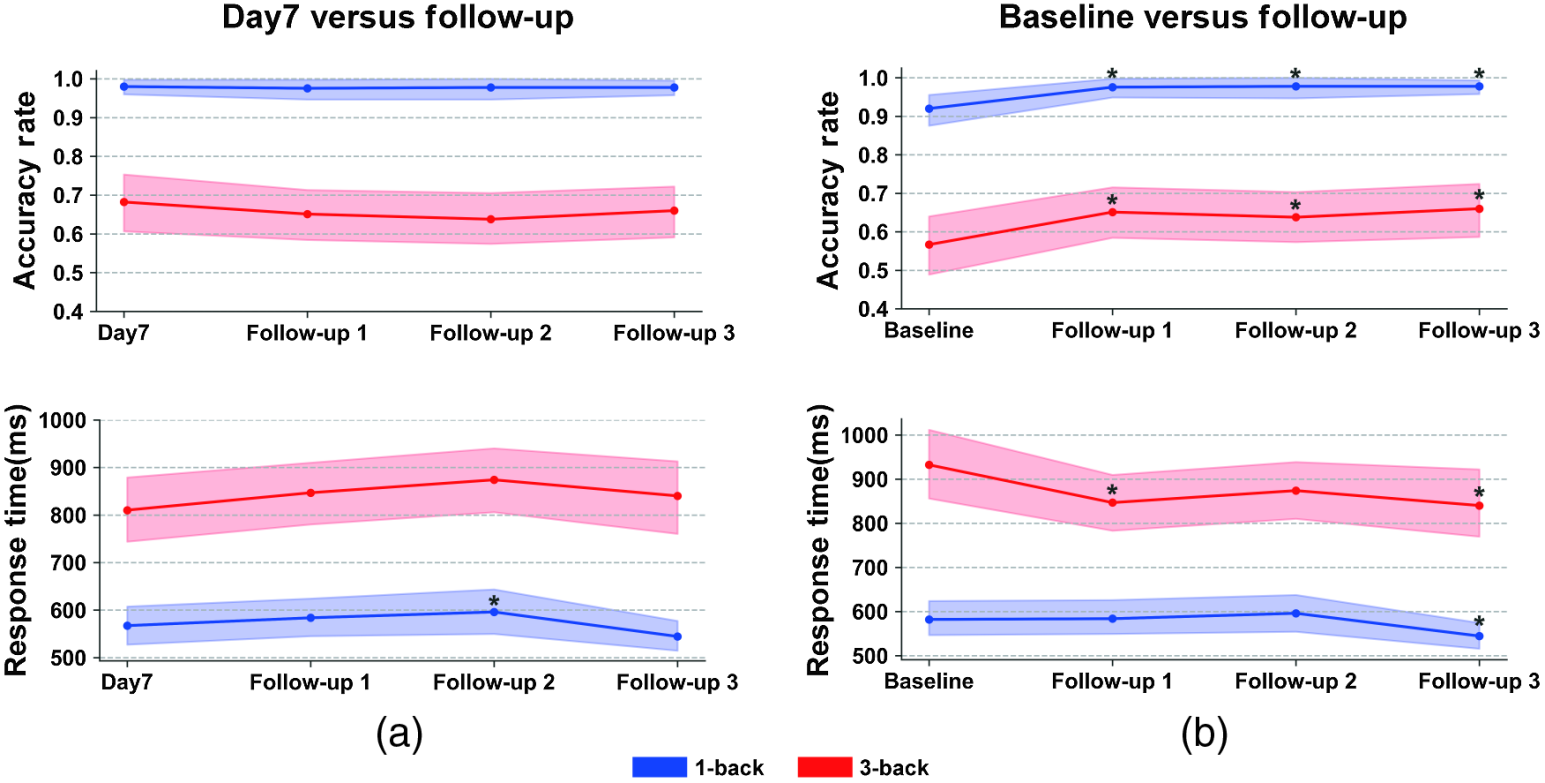
The persistent effects of repeated tPBM treatment on working memory. (a) Comparison between follow-ups and day 7 on accuracy rate and response time. (b) Comparison between follow-ups and baseline on accuracy rate and response time. The shaded area represents the standard error of the mean. *p<0.05.

A one-way ANOVA comparing the three follow-ups with day 7 found no significant main effect of time for the 1-back condition [F(1.90,45.58)=0.048, p=0.95] or the 3-back condition [F(3,72)=0.97, p=0.41] in terms of accuracy rate or for the 3-back condition [F(3,75)=1.78, p=0.16] in terms of response time. Yet, the response time in the 1-back condition was found to have a significant main effect of time [F(3,75)=4.05, p=0.01]. Specifically, the response time at follow-up 2 was significantly higher than at day 7 [F(1,25)=4.29, p=0.05]. In general, these findings suggest that the effect of repeated tPBM treatment on these behavioral metrics did not decline during the three weeks [Fig. 3(a)].

When the three follow-ups and the baseline were compared, a significant main effect of time was found on accuracy rate and response time in 1- and 3-back conditions [accuracy rate: 1-back: F(1.74,41.76)=4.19, p=0.03; 3-back: F(3,72)=3.70, p=0.02; response time: 1-back: F(2.35,58.76)=4.34, p=0.01; and 3-back: F(3,75)=3.48, p=0.02]. Specifically, the pairwise analysis revealed significant increases in accuracy rate throughout the three Follow-ups in both conditions [Fig. 3(b), 1-back: follow-up 1: F(1,24)=4.35, p=0.05; follow-up 2: F(1,24)=5.19, p=0.03; follow-up 3: F(1,24)=7.14, p=0.01; 3-back: follow-up 1: F(1,24)=5.21, p=0.03; follow-up 2: F(1,24)=4.60, p=0.04; and follow-up 3: F(1,24)=7.32, p=0.01]. For the response time, it was found to be significant decrease in the 3-back condition for the follow-up 1 [F(1,25)=4.98, p=0.04) and the follow-up 3 [F(1,25)=6.65, p=0.02] and marginally significant decrease for the follow-up 2 [F(1,25)=3.10, p=0.09) [Fig. 3(b)]. Response time also significantly decreased at Follow-up 3 in 1-back [Fig. 3(b), F(1,25)=4.80, p=0.04].

### 3.3 Exclusion of Practice and Placebo Effects

As indicated in Table 2, the comparisons (three follow-ups versus day 7; three follow-ups versus baseline) revealed no significant difference among the 1-, 2-, and 3-back conditions for either accuracy rates or response times (p>0.05, corrected). The results suggest that the placebo effects and the practice effects from repeated exposure to the working memory task did not bring about behavioral improvement on participants.

**Table 2:** Working memory performance of the control group. The behavioral performance was compared between follow-up and day 7 as well as follow-up and baseline. The mean and standard deviation of the behavioral performance during follow-ups were shown. The participants who completed the same experimental conditions were included in the statistical comparisons.

			Follow-ups versus day 7			Follow-ups versus baseline		
			Follow-up 1	Follow-up 2	Follow-up 3	Follow-up 1	Follow-up 2	Follow-up 3
Accuracy rate	1-back	Mean (std)	0.83 (0.18)	0.91 (0.14)	0.95 (0.08)	0.83 (0.18)	0.90 (0.14)	0.96 (0.07)
		*p*	0.28	0.26	0.06	0.12	0.47	0.18
	2-back	Mean (std)	0.70 (0.19)	0.74 (0.23)	0.78 (0.14)	0.70 (0.20)	0.76 (0.22)	0.78 (0.15)
		*p*	0.23	0.47	0.37	0.38	0.31	0.25
	3-back	Mean (std)	0.47 (0.24)	0.60 (0.22)	0.60 (0.26)	0.48 (0.23)	0.61 (0.20)	0.61 (0.22)
		*p*	0.48	0.08	0.17	0.36	0.31	0.45
Response time (ms)	1-back	Mean (std)	578.67 (118.27)	576.51 (146.26)	520.47 (122.14)	580.42 (109.85)	581.57 (136.46)	526.88 (106.26)
		*p*	0.145	0.30	0.06	0.30	0.34	0.19
	2-back	Mean (std)	662.03 (174.18)	650.73 (126.04)	720.66 (198.00)	667.58 (138.20)	660.55 (120.69)	708.46 (172.05)
		*p*	0.17	0.12	0.09	0.31	0.18	0.50
	3-back	Mean (std)	702.29 (208.49)	735.26 (192.05)	796.21 (151.67)	722.17 (200.70)	740.02 (189.73)	790.59 (132.99)
		*p*	0.50	0.43	0.18	0.49	0.50	0.43

## 4 Discussion

This randomized and sham-controlled experimental study examined whether seven-day repeated tPBM treatment, compared to single tPBM treatment and baseline, led to better N-back working memory performance in healthy older adults and evaluated the persistent effects of repeated tPBM treatment. In general, the current study endorsed the theory that seven-day repeated tPBM treatment resulted in improved behavioral outcomes of working memory performance (e.g., accuracy rate) in more difficult tasks (i.e., 3-back tasks). More importantly, such behavioral improvements were found to persist for three weeks following repeated tPBM treatment.

As a novel and non-invasive technique for improving brain function, tPBM has been demonstrated to improve cognition in both animals^17^^–^^19^ and human subjects,^4^^,^^7^^,^^20^ ranging from young college students to elderly individuals. Most of these studies from human participants have primarily focused on patients,^11^^,^^21^ utilizing either single^4^ or repeated^13^ tPBM treatment and witnessing improvement in working memory^7^, sustained attention,^10^ or the other cognitive functions.^8^^,^^9^ However, there were currently few studies on healthy older adults and no reports evaluated the difference between single and repeated tPBM treatment on enhancing cognition. This current study aimed to bridge this gap by examining the effect of single and seven-day repeated tPBM treatment on working memory performance in healthy older adults. Meanwhile, this study considered that previous studies on tPBM-induced effects on memory improvement mainly used DMS tasks,^7^^,^^13^^,^^22^ without clarifying that tPBM treatment might vary according to cognitive loads triggered by the task designs. Here, we adopted an N-back task with three levels of difficulty (i.e., N=1,2,3) and specifically investigated whether tPBM treatment produced discriminative outcomes on the improvement of working memory, as completing a 3-back task requires more cognitive resources than 1- or 2-back task.^23^

As expected, significant improvements in working memory performance in both 1-back and 3-back tasks were observed after single or seven-day repeated tPBM treatment (Fig. 2). This finding indicated that tPBM treatment with different frequencies played active roles in overall memory improvement. This finding was consistent with several previous studies reporting that cognitive function, e.g., memory,^7^ attention,^10^ and executive function^9^ were improved after tPBM treatment. Recently, one study from Chan et al.^24^ specifically examined the hemodynamic changes during 0- and 3-back working memory tasks in young healthy adults after frontal tPBM stimulation (one duration for 350 s, three sections). The authors observed a significant reduction in frontal hemodynamic activation in the experimental group during the difficult task, i.e., the 3-back condition. The finding provided neural evidence for improvements in working memory as performing difficult tasks. As such, both a previous brain imaging study^24^ and our current behavioral investigation support that tPBM reduces the cognitive efforts needed to complete tasks with high memory loads. Notably, for the 2-back task, the current study did not observe significant changes in the behavioral outcomes after single or seven-day repeated tPBM treatment. However, it is necessary to point out that the trend of cognitive improvement was still observed in the 2-back condition (Fig. 2). As an emerging neuromodulation technique, whether the tPBM treatment takes effect better for more difficult or much easier tasks compared to the intermediate ones remains unknown and needs to be further explored in the future.

Although both single and repeated tPBM treatment resulted in significant improvements in working memory performance, we also found that receiving a seven-day repeated tPBM treatment resulted in a higher accuracy rate in 1-and 3-back tasks than receiving only a single tPBM treatment (Fig. 2). We therefore chose the repeated treatment to investigate the persistent efficacy of tPBM. Meanwhile, we considered that the older individuals have thicker extracerebral tissues (ECT) negatively correlated with tPBM energy deposition, which necessitates repeated stimulations with a larger number of photons interacting with the tissues.^14^ Actually, several studies from both animals and human subjects demonstrated that repeated tPBM treatment have a positive effect on the improvement of cognitive function.^13^^,^^17^^,^^19^ For example, Tanaka et al.^17^ conducted an animal experiment in which chronic tPBM treatment was administered to the head of rats (3 min daily for ten days) and found improvement in anxiety and depression-related behaviors compared to those with a single exposure (3 min). Furthermore, with a human participant study, Vargas et al.^13^ found that chronic tPBM treatment (five weekly sessions, 8 min each) improved working memory and attention performance better than a single stimulation (8 min). As such, these combined studies demonstrate that the adequate number of photons interacting with tissue through repeated stimulation could actively elicit the photodissociation of nitric oxide from CCO, enhance the adenosine triphosphate production and frontal oxygen consumption and metabolic capacity, and ultimately benefit brain functions for cognitive processing.^7^

To take it a step further, we also speculate that tPBM treatment could change the functional network architecture in the brain that facilitates the improvement of working memory in older adults in the current study. This point of view was supported by studies from a widely used neuromodulation technique, e.g., transcranial direct current stimulation (tDCS).^25^^–^^29^ For example, Reinhart and Nguyen^25^ found an increase in temporal integration within large-scale cortical networks in older adults after 25 min of stimulation, which greatly boosted working memory performance in older adults. Furthermore, Meinzer et al.^26^ also found that a continued 20 min of tDCS stimulation could reverse age-associated cognitive declines in healthy elderly adults, leading to a significant reduction of task-related hyperactivity and a more youth-like cortical connectivity pattern. Intriguingly, several recent studies from tPBM have also found that functional connectivity of large-scale brain networks was changed in the regions of the frontal and parietal cortex associated with memory processing.^8^^,^^30^^,^^31^ Therefore, it is reasonable to speculate that functional connectivity in the regions related to working memory could also be modulated by repeated tPBM stimulation and such changes would possibly contribute to cognitive improvement in older adults in the current study. However, whether the seven-day repeated tPBM treatment could lead to an optimally integrated network organization that facilitates working memory improvement need to be further explored with neuroimaging techniques in the future.

Another essential finding in this study is that the group that received repeated tPBM treatment showed significantly enhanced working memory performance (i.e., improved accuracy rate and response time in 3-back tasks) for three weeks following the intervention [Fig. 3(b)]. We speculate that the general persistence of improvement in working memory performance could be attributed to the lasting effects of tPBM treatment, which is in line with several previous studies.^7^^,^^9^^,^^32^ For instance, a study^7^ found significant benefits in positive and negative affective states of healthy volunteers two weeks after a single tPBM treatment compared to the affective states of the sham group. Another study^33^ harnessed similar treatment in patients with mild traumatic brain injury for 18 sessions (three sessions per week for six weeks) and found increased cognitive performance in patients for one week, one month, and two months following the intervention.

Notably, the current study included a control group (Fig. 1) to exclude influences of the placebo effects from sham treatment and the practice effects from repeated exposure in behavioral tasks to healthy older adults. The results showed that either accuracy rate or response time had no significant difference between follow-ups and day 7 or between follow-ups and baseline in the 1-, 2-, and 3-back conditions (Table 2). Although it was noticed that the behavioral performance showed a trend toward an increase over sessions, e.g., the accuracy rate for 1- and 2-back conditions increased during follow-ups (Table 2), such an increase in behavioral outcomes did not result in significant changes in the N-back task. These results from the control group suggest that the repeated exposure in the working memory task did not bring about obvious habituation effects on the participants. Regarding this issue, previous documents have indicated that cognitive training with sufficient frequency and intensity, e.g., lasting ∼30  min or more each day for several days, led to practice effects (or the improvement in cognition) in older adults.^2^^,^^34^^,^^35^ As such, our current findings from the control study further support that the improved working memory performance in the experimental group on the older adults was from the active tPBM stimulation.

It is also worth noting that light power is an essential parameter in tPBM treatment research. The biphasic dose-response, or bell-shaped curve, in tPBM treatment, has been shown both in in vitro studies and in animal experiments,^36^ which indicates that inappropriate dosimetric parameters may lead to negative results and that the optimal dose will result in optimal behavioral performance.^32^^,^^36^^,^^37^ Skopin and Molitor^38^ used a 980-nm laser with increasing irradiance (control, 26, 49, 73, 97, and $120\;\mathrm{mW}/{\mathrm{cm}}^{2}$) on a scratch wounded culture of human fibroblasts and found that low- and medium-dose laser light accelerated cell growth, whereas high-intensity light negated the beneficial effects of laser exposure. Similarly, the dose of the single tPBM treatment in this study might not be enough to maximize the effect, whereas repeated tPBM treatment might have ensured that the dose was sufficient to achieve the optimal outcome, and thus one week of stimulation produced a better accuracy rate in the N-back task compared to a single stimulation. The current study only had seven days of tPBM treatment to avoid any decreased effects of excessive laser exposure. However, the optimal dose of tPBM treatment for cognitive improvement in healthy older adults or other participant populations needs further exploration before practical applications of tPBM treatment can be implemented.

Although the results are encouraging, the present study has several limitations. An obvious limitation is that some participants dropped out halfway for various personal reasons, which decreased the sample size in the following sessions. Although participants will inevitably drop out of studies midway during a longitudinal study, we hope that future studies will be able to recruit many more participants to confirm our current findings. Furthermore, the current results are limited to behavioral investigations, and further brain imaging and neurophysiological evidence (e.g., changes in CCO) are needed to support the present findings. In addition, the efficacy evaluation of single and seven-day repeated tPBM was based on one group of participants. Ideally, groups of single and repeated treatment should be separated to prevent possible habituation effects. Finally, this study did not examine the persistent effect of single tPBM treatment on improving working memory performance, so it is impossible to infer the duration of effects from single tPBM treatment or to compare differences in duration between repeated and single tPBM treatment.

## 5 Conclusion

We examined the effect of repeated tPBM treatment, compared to single tPBM treatment and baseline, on working memory performance in healthy older adults and evaluated the persistent effects of repeated tPBM treatment. Our results demonstrated that repeated tPBM treatment led to more significant working memory improvements for relatively easy and more challenging tasks in healthy older adults and such improvement was sustained for at least three weeks after stimulation. These findings suggest that repeated tPBM treatment has potential therapeutic applications for enhancing memory performance in healthy older individuals.

## Data Availability

The data presented in this study are available on request from the corresponding author since we have not set up a public archive platform for data sharing.
